# Ag_2_O Nanoparticles as a Candidate for Antimicrobial Compounds of the New Generation

**DOI:** 10.3390/ph15080968

**Published:** 2022-08-05

**Authors:** Sergey V. Gudkov, Dmitriy A. Serov, Maxim E. Astashev, Anastasia A. Semenova, Andrey B. Lisitsyn

**Affiliations:** 1 Prokhorov General Physics Institute of the Russian Academy of Sciences, 119991 Moscow, Russia; 2 V. M. Gorbatov Federal Research Center for Food Systems of Russian Academy of Sciences, 109316 Moscow, Russia

**Keywords:** silver oxide, nanoparticles, bacteriostatic effect, bactericidal effect, fungistatic effect, fungicidal effect, mammalian cells cytotoxicity, green synthesis, new materials development

## Abstract

Antibiotic resistance in microorganisms is an important problem of modern medicine which can be solved by searching for antimicrobial preparations of the new generation. Nanoparticles (NPs) of metals and their oxides are the most promising candidates for the role of such preparations. In the last few years, the number of studies devoted to the antimicrobial properties of silver oxide NPs have been actively growing. Although the total number of such studies is still not very high, it is quickly increasing. Advantages of silver oxide NPs are the relative easiness of production, low cost, high antibacterial and antifungal activities and low cytotoxicity to eukaryotic cells. This review intends to provide readers with the latest information about the antimicrobial properties of silver oxide NPs: sensitive organisms, mechanisms of action on microorganisms and further prospects for improving the antimicrobial properties.

## 1. Introduction

Since the moment of their discovery, antibiotics have been the “golden standard” in the treatment of many bacterial infections [1,2]. Unfortunately, the uncontrolled use of over-the-counter (OTC) antibiotics available without prescription has led to the emergence of new antibiotic-resistant bacterial strains. Diseases caused by such bacteria are not amenable to treatment. This phenomenon is called antibiotic resistance [3,4,5]. The development of antibiotic resistance in bacteria led to a new wave of growth in the number of infectious diseases and the necessity to search for new antimicrobial agents [6]. One of the ways to overcome antibiotic resistance in bacteria is the use of metal and metal oxide nanoparticles (NPs) [7]. Fungal diseases are a multi-national problem. More than 150 million people in the world have severe fungal diseases. More than 1.5 million cases of fungal diseases have a lethal outcome [8]. The problem is exacerbated by the development of fungal resistance to antifungal drugs [9]. There are reports about the antifungal properties of metal oxide NPs [10,11]. Since the beginning of the COVID-19 pandemic, special attention has been given to the search for inexpensive and effective antiviral agents [12,13].

The antimicrobial properties of silver and its compounds have been known since ancient times. The first references to the use of silver are dated back to 3500–1000 B.C. In particular, silver was used for dishware production and water storage; later on, there were attempts to use silver powder to treat various diseases [14,15,16]. It has been shown many times in the literature that nanoparticles (NPs) of silver and its compounds have significant bactericidal, fungicidal and antiviral activities [17,18,19]. Ag_2_O NPs have attracted particular attention of researchers in the field of nanomaterials because of their unique properties that ensure multiple functions and a wide field of application. The most significant applications of Ag_2_O NPs are the production of catalyzers, chemical sensors, optoelectronic devices and systems of targeted delivery of drugs in vivo [20,21,22,23,24]. Ag_2_O NPs also have significant antimicrobial potential [25,26,27]. Silver oxide is used as an antimicrobial agent in the creation of biocompatible materials when developing bone implants [28]. Biomedical applications also include cancer therapy, wound treatment, tissue protection from oxidative stress, therapy of stomach ulcer, etc. [29,30,31]. An important application at the interface of biomedicine and ecology is the use of Ag_2_O NPs for photocatalytic destruction of pharmaceutical micro-pollutants [32].

The aim of this review is to provide readers with methods for Ag_2_O NP production, a range of sensitive microorganisms, mechanisms of the antimicrobial activity and some ways for improving their antimicrobial properties.

## 2. Sensitive Microorganisms

**Figure 1:**
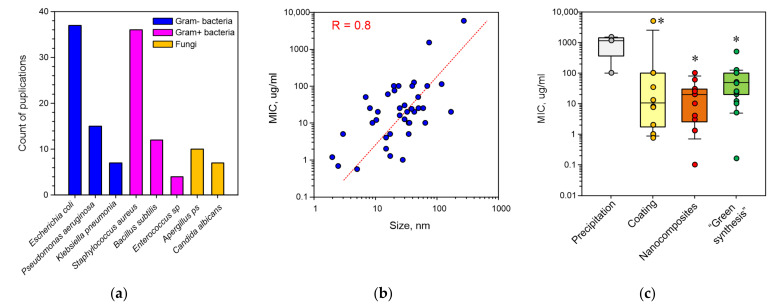
Results of the data analysis regarding antimicrobial properties of Ag_2_O NPs: (**a**) microorganisms, against which the inhibitory activity of NPs was shown most often; (**b**) dependence of MIC against *E. coli* on NP sizes. R—value of the correlation coefficient; (**c**) dependence of MIC on a method of NP generation. *—*p* < 0.05, a significant difference from the precipitation variant using the Mann–Whitney test. Each dot represents a mention in one publication. The data are presented as medians, percentiles (10, 25, 75 and 90%).

There are data in the literature about the antimicrobial activity of Ag_2_O NPs against, at least, 53 microbial species (Table 1), including 21 species of Gram-negative bacteria, 15 species of Gram-positive bacteria and 17 fungal species (Figure 1a). Among the most often mentioned organisms are Gram-negative bacteria *Escherichia coli*, *Pseudomonas aeruginosa* and *Klebsiella pneumoniae*; Gram-positive bacteria *Staphylococcus aureus* and *Bacillus subtilis*; and fungi *Aspergillus* and *Candida albicans*. All mentioned microorganisms have epidemiological significance. Antibiotic-resistant strains are most often found among *Escherichia coli* and *Staphylococcus aureus* [27,33,34,35,36]. We expected that the antimicrobial activity of Ag_2_O NPs against bacteria with different structures of cell wall (Gram-negative and Gram-positive) will greatly differ. An approximately equal amount (~20) of species of Gram-negative bacteria and Gram-positive bacteria sensitive to Ag_2_O NP was observed. This fact suggests the universality of the mechanisms of the antibacterial activity of Ag_2_O NPs. Ag_2_O NPs not only effectively inhibited bacterial growth, but also killed them. Therefore, Ag_2_O NPs are a perfect candidate for the role of a therapeutic agent against nosocomial bacterial infections [37].

When assessing a ratio of reports about the bactericidal and bacteriostatic activity of Ag_2_O NPs (Table 1), we found that bacteriostatic activity was described in about 75% of studies and bactericidal activity in 25% of studies. It is worth noting that the ratio of reports about the bactericidal and bacteriostatic activity of Ag_2_O NPs (equal to 1:3) is comparable to other widely used metal oxide NPs with antimicrobial activities, for example, iron oxides or ZnO NPs [7,88]. Iron oxides or ZnO NPs demonstrated high cytotoxicity in contradistinction to Ag_2_O NPs [89,90,91]. Having the same antimicrobial activity with other metal oxide NPs and low cytotoxicity makes Ag_2_O NPs an interesting candidate for the role of new generation antiseptic. For antifungal activities, the ratio shifted towards a reduction of the fungicidal activity. Only 15% of studies indicate the presence of the fungicidal effect and 85% contain data about the fungistatic effect. Therefore, fungi have higher resistance to Ag_2_O NPs compared to bacteria. This effect can be explained by the higher resistance of eukaryotic cells to the genotoxic effect of metal ions compared to prokaryotes, in particular, due to differences in the structure of the genetic apparatus and function of the reparation systems [92,93,94].

## 3. Synthesis Methods

Methods for the synthesis of Ag_2_O nanoparticles can be divided into physical, chemical and biological, otherwise referred to as “green synthesis” [95].

Chemical methods include various types of precipitation. The simplest method is realized when mixing AgNO_3_ with NaOH at high temperatures [13,58,75,96].

In this case, NP synthesis occurs in two stages described by the reaction equations:

AgNO_3_ + NaOH → AgOH + Na^+^ + NO_3_^−^ (1)

2AgOH → Ag_2_O + H_2_O (pK = 2.875) (2)

Modifications of the method are possible: the addition of strong oxidizers, for example, K_2_S_2_O_4_, and KOH as a base [19,50]. Sometimes AgNO_3_ is obtained directly at the moment of synthesis upon the oxidation of silver foil with nitric acid; then, precipitation with alkali described above is performed [77]. To prevent the premature aggregation of synthesized Ag_2_O NPs, a surfactant—for example, citrate, polyethylene glycol, triethylene glycol, chitosan, urea and other compounds—can be added to the reaction mixture [40,82,96,97,98,99]. Another method for Ag_2_O NP production is the reduction of AgNO_3_ using organic acids citrate, acetate and oleic acid [45,53,56]. In the literature, this method is sometimes called the sol-gel method [100]. A method of Ag_2_O production upon the reduction of complex compounds, for example, ammoniate [Ag(NH_3_)_2_]_x_, is described [59,101]. To obtain NPs with a complex chemical composition, the drying of metal oxide NPs in the AgNO_3_ solution is used, as in the case of TiO_2_/Ag_2_O NPs [47].

The electrochemical synthesis (anode oxidation of metal silver) [102], precipitation upon ultrasound treatment [63], boiling [67,78], treatment with microwave radiation [22,78], evaporation of metal silver under the action of plasma [81] and laser ablation in water [52,53] can be assigned to physical methods.

Chemical and physical methods used today for NP synthesis can be expensive, require high temperatures and pressure or lead to the generation of waste that is hazardous for the environment [103]. Therefore, biological methods for the synthesis of nanomaterials, the so-called “green synthesis”, are preferable [26,104]. Moreover, silver oxide NPs obtained using biological methods have several advantages: low cost of synthesis, high antimicrobial activity, low cytotoxicity to mammalian cells and the possibility to use in pharmacology and biomedicine, like for NPs obtained by classical methods [105]. Similar to Ag NPs, “green” synthesis using extracts of medicinal plants is one of the methods for improving the antimicrobial properties of Ag_2_O NPs [106].

“Green synthesis” of Ag_2_O NPs consists of, as a rule, the reduction of water-soluble salt AgNO_3_ in an extract of medicinal plants or cultural liquid of non-pathogenic/weakly pathogenic microorganisms [107,108,109].

However, cases of real biosynthesis of Ag_2_O NPs are described, for example, synthesis by bacteria isolated from seeds of agricultural crops and cultivated in medium with the addition of AgNO_3_ [110,111] and soil bacteria *Nitrobacter* sp. [61]. In addition, methods for synthesis of Ag/Ag_2_O NPs by silver reduction in the medium of *Fusarium oxysporum* mycelium or dead biomass of yeasts [56,80] were described.

## 4. Methods for Studying Ag_2_O NPs

Dozens of methods have been applied to describe the parameters of Ag_2_O NPs. These methods are commonly used to study other Me/Me_x_O_y_ NPs [26]. To determine the size and shape of Ag_2_O NPs, various microscopic methods are used: atomic force microscopy (AFM) [112], scanning tunneling microscopy (STM) [113], scanning electron microscopy (SEM) [114] and transmission electron microscopy (TEM). The indicated methods allow us to image dry NPs and assess their size, shape, distribution on the surface of composite materials. To assess the elementary composition, proportion of organic impurities and conjugates, the following methods are used: UV–vis spectroscopy [115], Fourier transform infrared spectroscopy (FT-IR) [116,117], energy dispersive spectroscopy (EDX) [118], X-ray photoelectron spectroscopy (XPS) [119] and thermal gravimetric analysis (TGA) [120].

To determine the crystalline structure of NPs, the X-ray diffraction (XRD) method is applied [121,122]. To assess the hydrodynamic radius of NPs and stability of NP colloids in solvents, the dynamic light scattering (DLS) method and measurement of zeta potential, respectively, are used [123]. Assessment of the NP surface area and rheological properties of obtained nanomaterials is carried out by differential scanning calorimetry (DSC) and the Brunauer–Emmett–Teller (BET) method, respectively [124,125]. In the case of NP embedding into a polymeric material, it is possible to assess NP spatial distribution inside a polymeric matrix using modulation interference microscopy (MIM) [126].

## 5. Mechanisms of the Antimicrobial Activity

**Figure 2:**
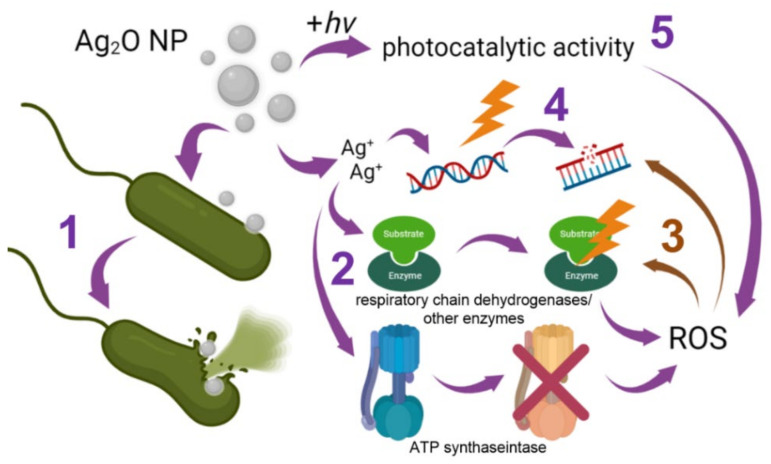
Schematic representation of mechanisms of the antibacterial activity of Ag_2_O NPs (explanations are given in the text).

Antimicrobial properties of NPs are conditioned, first of all, by the antimicrobial properties of elements being their constituents. Silver ions show high toxicity to microorganisms. For example, Ag^+^ causes the death of *Aspergillus niger* spores at a concentration of 5.5 × 10^−5^ M (0.00006% *w/w*) and higher [127]. Ag NPs exert a significant antibacterial effect beginning from a concentration of 20 µg/mL [128,129]. It is shown that silver can be accumulated in microorganisms as Ag^0^, Ag_2_O or Ag^+^ [130]. Five mechanisms (as a minimum) of the antibacterial activity are described for these forms (Figure 2) [131].

The first mechanism is binding to the bacterial cell wall and disruption of the cell wall integrity, resulting in direct damage of the cell envelope and cytoplasmic components [96,97,100]. It is assumed that after Ag_2_O NP penetration into a bacterial cell, the release of Ag^0^ and/or Ag^+^ having the bactericidal activity according to the mechanisms described below takes place [132,133].

The second mechanisms of toxicity is binding to SH-groups of proteins with the subsequent disorder of their function [134]. Silver-induced inactivation of bacterial enzymes, in particular, dehydrogenases of the respiratory chain, is described [110]. This, in turn, inhibits ATP synthesis, disturbs the energy balance in cells, enhances an intracellular ROS production and causes oxidative stress [110,135]. Moreover, Ag_2_O NPs are able to release O_2_, which can also exert antibacterial activity [96].

The third mechanism is the oxidative stress described above. ROS cause protein modifications and exert a genotoxic effect [136,137,138]. An increase in ROS generation leads to the destruction of the cell wall and biofilms of both Gram-positive and Gram-negative bacteria [123].

The fourth mechanism of the antibacterial activity of Ag_2_O NPs is the genotoxic activity of Ag compounds, which after penetration inside a bacterial cell interact not only with proteins but also with phosphoric acid residues in DNA molecules [59,139].

It is assumed that silver compounds from Ag_2_O NPs and Ag NPs are also capable of binding to the N7 atom of guanine in DNA, therefore disturbing the process of its replication, inhibiting cell division [139].

The fifth mechanism is photocatalytic activity. The addition of Ag_2_O NPs can enhance the photocatalytic properties of other metal NPs. In particular, composites of Ag_2_O/TiO_2_ NPs and Ag_2_O/ZnO NPs demonstrate enhanced photocatalytic activity compared to TiO_2_ or ZnO NPs [140,141,142]. Furthermore, photocatalytic activity of Ag_2_O NPs was demonstrated. It is interesting that the photocatalytic activity of Ag_2_O NPs enhanced after the conjugation of Ag_2_O NPs with certain pharmaceutical agents, for example, moxifloxacin [48,62].

It is notable that Ag_2_O NPs possess high toxicity to pathogenic microorganisms and low toxicity to soil microorganisms. In particular, soil *Nitrobacter* sp., *Bacillus* sp. and *Pseudomonas* strains are able to synthesize Ag_2_O NPs from AgNO_3_ in amounts sufficient for the growth inhibition of pathogenic microorganisms of the human oral cavity [49,54,61,78,143]. Specific Ag_2_O NP cytotoxicity to pathogenic microorganisms is an attractive feature for the creation of eco-friendly antimicrobial materials and preparations.

## 6. Methods for Improving Antimicrobial Properties

In meta-analysis, we found a dependence of the bacteriostatic activity (expressed in MIC) on NP size (Figure 1b). When a NP’s size decreases, an increase in its toxicity to microbes is observed. This dependence corresponds to the literature data about NPs of other metal oxides [7,144], and can be explained by a growth in the release of Ag^+^, Ag^0^ and Ag_2_O from NPs into the surrounding solution due to an increase in the area to volume ratio.

Antimicrobial properties of Ag_2_O NPs can be improved at the initial stage of NP synthesis: precipitation of Ag_2_O NPs. For example, precipitation of Ag_2_O NPs in medium with low (10 mM) or high (100 mM) concentration of AgNO_3_ lead to obtaining cubic or octahedral Ag_2_O NPs, respectively [74]. Cubic Ag_2_O NPs showed more pronounced bacteriostatic effects compared to octahedral [74].

The most common other modifications of Ag_2_O NP synthesis are NP coating with polymers, Ag_2_O NP inclusion into other nanocomposites or fusion with NPs of oxides of other elements and NP synthesis in the medium of a substrate of the biological origin—most often an extract of plant leaves (Figure 1c) [34,47,118].

Coatings can be conditionally divided into two large groups. The first group includes organic polymers: chitosan, polyethersulfone, cellulose acetate, polyvinyl alcohol, polyethylene terephthalate and starch [41,42,43,57,96]. This modification commonly had bacteriostatic and fungistatic activity [39,43]. Pharmaceutical preparations, in particular, aspirin and moxifloxacin, can be assigned to the second group [43,62]. For example, Ag_2_O NP coating with aspirin increased their bacteriostatic and fungistatic activity by 50% compared to non-conjugated NPs. In the case of Ag_2_O NP conjugation with moxifloxacin, a more pronounced increase in the bacteriostatic and fungistatic activity of Ag_2_O NPs (by 2–3 times) was shown [62]. Ag_2_O NP coating with chitosan allows practically 100% inhibition of the bacterial growth to be achieved irrespective of their Gram stain group [40]. An opportunity to use conjugates chitosan/Ag_2_O NPs for the creation of fabrics and cloths with the bacteriostatic properties is shown [40,41].

Examples of nanocomposites with Ag_2_O NPs are relatively rare. Among them, composites with ZrO_2,_ TiO_2_ NPs, H_2_Ti_3_O_7_·2H_2_O and graphene oxide can be highlighted [60,122,123]. The addition of graphene oxide resulted in a dose-dependent increase in the antibacterial properties of Ag_2_O NPs. It is notable that in the case of graphene oxide, an enhancement of the bacteriostatic properties against Gram-negative bacteria was more pronounced [46].

The most common modification of Ag_2_O NP synthesis is the so-called “green synthesis”. There are reports about the use of extracts of plants *Abroma augusta, Lawsonia inermis, Ficus benghal, Lippia citriodora, Eupatorium odoratum, Cleome gynandra, Aloe vera, Vaccinium arctostaphylos, Coleus aromaticus, Rhamnus virgate, Cyathea nilgiriensis, Centella Asiatica*, *Tridax* sp., *Hylocereus undatus, Paeonia emodi, Pinus longifolia* and *Telfairia occidentalis Telfairia occidentalis* [33,34,35,36,37,51,60,64,65,66,69,73,76,83,84,85]; fungi *Fusarium oxysporum, Kitasatospora albolonga, Rhodotorula mucilaginosa* and *Aspergillus terreus* VIT 2013 [27,78,79,135]; and culture media of bacteria *Bacillus paramycoides, Bacillus thuringiensis* SSV1, *Nitrobacter* sp. (strain NCIM 5067) and *Pseudomonas aeruginosa* M6 [63,77,113,114]. “Green synthesis” enables Ag/Ag_2_O NPs to be obtained from wastes of silver mines, which may increase the production of silver mines and decrease environmental pollution [145]. “Green synthesized” Ag_2_O NP had not only bacteriostatic activity, but also fungicidal activity [37,79,125].

It is worth noting that all modifications of Ag_2_O NP synthesis enhance their antimicrobial properties compared to the chemical synthesis methods, in particular, precipitation (Figure 1c). Therefore, the selection of the conditions of Ag_2_O NP synthesis can make it possible to obtain NPs with high antimicrobial activity against antibiotic resistance bacteria. There are data that show that a synergetic effect is possible due to the use of several methods to improve the bacteriostatic activity of Ag_2_O NPs [75], for example, the synthesis of complex composites Cu·PES/CA/Ag_2_O NPs. This composite had more pronounced bacteriostatic properties compared to PES/CA/Ag_2_O NPs [42].

A growth in the studies devoted to the creation of various composites with the addition of Ag/Ag_2_O NPs (Table 1) allows us to suggest that the development of new composite materials with Ag_2_O NP introduction and, as a consequence, the extension of application fields for Ag_2_O NP-based nanomaterials will be promising investigations in this field [60,118,122,123].

## 7. Cytotoxicity to Human Cells

Data on Ag_2_O NP cytotoxicity are ambiguous and constantly being enriched. There are data about the toxicity of Ag_2_O NPs/*Aspergillus terreus* to Dalton’s lymphoma ascites (DLA) cells, which enables the use of Ag_2_O NPs in the therapy of oncological diseases [36]. High cytotoxicity of Ag_2_O/Ag NPs reported against breast cancer cell line MCF-7 and lung cancer cell line A549. Mechanisms of toxicity are genotoxic effects and ROS overproduction and membrane disruption [146]. Cytotoxicity of Ag NPs and consequently Ag_2_O NPs against eukaryotic cells is actively studied. Induction of apoptosis and necrosis by Ag_2_O/Ag NPs was shown on lung cells lines A549, MRC-5, bronchial cells BEAS-2B and NIH3T3, 3D-cultures of human primary small airway epithelial cell, etc. [147,148,149,150,151]. The ways to increase the cytotoxicity of Ag NPs against cancer and decrease against normal cells have been researched [152]. An interesting approach is using different coating agents; for example, Ag NP cytotoxicity increases in range “PVP > citrate > plant extracts > without coating”, but in the case of PVP and citrate, increased predominantly anticancer activity [153].

However, many studies report the low cytotoxicity of Ag_2_O NPs to eukaryotic cells. For example, Ag_2_O NPs did not affect the survival and migration of 3T3 fibroblast cells [63]. It was shown for Ag/Ag_2_O NPs/*R. mucilaginosa* that the cytotoxic action against eukaryotic cells was realized at concentrations 4–10 times higher than the cytotoxic action against bacteria and fungi [80]. For nanocomposites based on borosiloxane and PLGA and Ag_2_O NPs, the high bactericidal activity was found at Ag_2_O NP concentrations from 1 μg/ml; with that, the survival and the proliferation rate of eukaryotic cells on the above mentioned composites was comparable to these parameters obtained on the culture plastic [52,53]. Low cytotoxicity allows Ag_2_O NPs to be used for wound healing [37].

We assume that the cause of high biocompatibility with eukaryotic cells in the majority of studies is the use of Ag_2_O NP conjugates and composites instead of “pure” Ag_2_O NPs. We also proposed that Ag_2_O is more biologically invert compared to pure Ag.

Metal oxide NPs were potential drug delivery systems. The moderate/low cytotoxicity of Ag_2_O/Ag NPs makes them a perfect candidate for drug delivery systems [154,155,156]. Ag_2_O/Ag NPs can be used in anticancer and antiviral therapy [157,158,159]. Ag_2_O/Ag NPs can also be used as a photoactivated drug delivery unit, for example, in the localized induction of bone regeneration [160].

## 8. Conclusions

A search for antimicrobial agents of the new generation that allow us to overcome bacterial antibiotic resistance is an important task for world public health. Candidates for such agents are Ag_2_O NPs. Over the last three years, the interest of researchers in Ag_2_O NPs has increased manifold. The reason for this is the high toxicity to Gram-positive and Gram-negative bacteria, including antibiotic resistance, as well as fungi having epidemiological significance. Moreover, Ag_2_O NPs are inexpensive and easy to produce, and the field of their possible application includes regenerative medicine, prosthetics, therapy of oncological diseases, as well as the development of a wide spectrum of materials with antimicrobial properties (textile and construction). Ag_2_O NP cytotoxicity to eukaryotic cells and nonpathogenic microorganisms is significantly lower than against human pathogens, which makes Ag_2_O NPs an attractive candidate for the role of an antimicrobial agent safe for humans and the environment. Extension of the list of composite materials with the addition of Ag_2_O NPs and, as a consequence, an increase in the number of application fields for Ag_2_O NP-based nanomaterials can be considered the expected outcomes of investigations in this field.

## Figures and Tables

**Table 1:** Antimicrobial properties of Polymers/Ag_2_O nanocomposites.

№	Composition	Particle Size, nm	Microorganism Strains	Effect	MIC/MBC	Results	Reference
1	Ag_2_O NPs coating on glass	~1500	*Pseudomonas aeruginosa* (DSM-9644), *Staphylococcus aureus* (ATCC no. 6538), *Staphylococcus aureus* (MA43300 methicillin-resistant), SARS-CoV-2 virus	Bacteriostatic Bactericidal Antiviral	1.18 mg/mL	Coating of glass surfaces with Ag_2_O NPs significantly reduced the titers of the SARS-CoV-2 virus on the treated surface after 1 and 24 h. Ag_2_O NPs caused the death of all studied bacteria after 1 h. The activities against Gram-negative bacteria were more pronounced.	[13]
2	AgO NPs	~170	*Staphylococcus aureus*	Bactericidal	20 µg/mL	The bactericidal action of AgO NPs realized via disruption of the bacterial cell wall integrity detectable by K^+^ leakage from cells, increased Ag content in cell walls and TEM data.	[19]
3	Ag_2_O NPs in Ag_2_O NPs/Ag sensor for detection of 4-nitrotoluene	80–90	*Escherichia coli,* *Staphylococcus aureus*	Bacteriostatic	100 µg/mL	Ag_2_O NPs showed bacteriostatic effect against both studied bacteria. The antimicrobial effect against Gram-positive bacteria is much higher.	[22]
4	Ag_2_O NPs synthesized in *Aspergillus terreus* VIT 2013 culture	500–1000 (TEM images)	*Staphylococcus aureus* methicillin resistant	Bacteriostatic	~23.2 mg/mL * (0.1 mM Ag_2_O)	Ag_2_O NPs inhibited growth of all studied antibiotic-resistant *S. aureus* strains.	[27]
5	Ag_2_O NPs synthesized in *Rhamnus virgate* extracts	110–120	*Aspergillus flavus,* *Aspergillus niger,* *Bacillus subtilis,* *Candida albicans,* *Escherichia coli,* *Fusarium solani,* *Klebsiella pneumonia,* *Mucor racemosus,* *Pseudomonas aeruginosa,* *Staphylococcus aureus*	Bacteriostatic Fungistatic	28.125–112.5 µg/mL	Antimicrobial activity significantly varied depending on the species of microorganism. Ag_2_O NPs decreased viability of HepG2 cell line and HUH-7 cancer cells at concentrations above 9 µg/mL. Using of ethanol extract to Ag_2_O NPs synthesis increased their antimicrobial activity.	[33]
6	Ag_2_O NPs synthesized in *Pinus longifolia* extract	1–100	*Bacillus subtilis,* *Escherichia coli,* *Staphylococcus aureus*	Bacteriostatic	25 µg/mL	Ag_2_O NPs/*P. longifolia* inhibited the growth of both Gram-positive and Gram-negative bacteria equally	[34]
7	Ag_2_O NPs synthesized in *Paeonia emodi* extract	38–86	*Bacillus subtilis,* *Escherichia coli,* *Pseudomonas aeruginosa,* *Staphylococcus aureus*	Bacteriostatic	0.125 µg/mL	Bacteriostatic action against Gram-negative bacteria was more pronounced. The mechanism of bacteriostatic action is a photocatalysis.	[35]
8	Ag_2_O NPs synthesized in *Cyathea nilgiriensis* extract	8–40	*Bacillus subtilis,* *Escherichia coli,* *Klebsiella pneumonia,* *Micrococcus luteus,* *Salmonella paratyphi,* *Staphylococcus aureus,* *Aspergillus niger,* *Candida albicans*	Bacteriostatic Fungistatic	~100 µg/mL	Ag_2_O NPs/*C. nilgiriensis* showed bacteriostatic, antifungal and antitumor activity.	[36]
9	Natural hydrogel from *Abroma augusta*/Ag-Ag_2_O NP with varying polyphenol concentrations of 50, 100, 150 and 200 μg/mL	20–40	*Bacillus cereus* MTCC 430, *C. albicans* MTCC 227, *Escherichia coli* MTCC 443, *Klebsiella pneumoniae* MTCC 7162, *Pseudomonas aeruginosa* MTCC 741, *Staphylococcus aureus* MTCC 96	Bacteriostatic Bactericidal Fungicidal	12.5/25 µg/mL 12.5/25 µg/mL 25/50 µg/mL 25/50 µg/mL 25/50 µg/mL	Maximal antimicrobial effect of nanocomposite was observed at 200 μg/mL polyphenol concentrations.	[37]
10	Ag_2_O NPs mixed with chitosan solution (1% *w/v* in 1% acetic acid) and dried	~5		№	~5.8 mg/mL (stock 0.1 M AgNO_3,_ was used [38]; 0.05 M Ag_2_O was synthesized and diluted twice to 0.025 M)	Chitosan/Ag_2_O NPs inhibited growth of all studied bacteria.	[39]
11	Chitosan/Ag_2_O NPs suspension	10–20	*Escherichia coli*, *Staphylococcus aureus*	Bacteriostatic	2 µg/mL	Treating of cotton fibers by chitosan/Ag_2_O NPs suspension reduced Gram-negative and Gram-positive bacterial growth up to 100%.	[40]
12	Chitosan/Ag_2_O NPs suspension	100–200	*Escherichia coli*, *Staphylococcus aureus*	Bacteriostatic	2 µg/mL	Treating of cotton fibers by chitosan/Ag_2_O NPs suspension reduced bacterial growth and did not change coefficient of friction of the treated fabric.	[41]
13	Polyethersulfone (PES)/cellulose acetate (CA)/Ag_2_O NPs nanocomposite and Cu·PES/CA/Ag_2_O NP membranes	20–100	*Escherichia coli*	Bacteriostatic	8 mg/mL	PES/CA/Ag_2_O NPs and Cu·PES/CA/Ag_2_O NPs composites inhibited bacterial growth up to 20–30 and 80–90%, respectively, during 12–24 h.	[42]
14	Aspirin conjugated Ag_2_O NPs coated by polyvinyl alcohol (PVA) or starch	-	*Apergillus niger*, *Citrobacter freundii*, *Curvularia lunata*, *Enterobacter aerogenes*, *Escherichia coli*, *Proteus vulgaris*, *Staphylococcus aureus,* *Vibrio cholera*, *Helmentiasporium maydis*, *Paecilomyces lilacinusby*, *Rhizopus nigricans*	Bacteriostatic, Fungistatic	10 µg/mL	Aspirin conjugated Ag_2_O NPs inhibited microbial growth above 40%. Coating of Aspirin/Ag_2_O NP by PVA or starch increased percent inhibition to 60%.	[43]
15	Bayerite underpinned Ag_2_O/Ag NPs incorporated PMMA films	-	*Acinetobactor baumannii* C78 and C80, *Pseudomonas aeruginosa* RRLP1 and RRLP2	Bacteriostatic	0.034 and 0.017 mg/mL	Bayerite Ag_2_O/Ag nanohybrid demonstrated antibacterial and antibiofilm activities against tested standard strains and clinical isolates.	[44]
16	Graphene oxide (GO)/Ag_2_O NPs composite	36.3–49.9	*Escherichia coli*, *Staphylococcus aureus*	Bacteriostatic	20 mg/mL	GO/Ag_2_O NPs composite was more effective against Gram-negative bacteria. Increasing of GO wt% improved bacteriostatic activity of nanocomposite.	[45]
17	Polyethylene terephthalate (PET)/Ag_2_O NPs composite	50–500	*Escherichia coli*	Bacteriostatic	-	PET/Ag_2_O NPs inhibited bacterial growth. Bacteriostatic was same in PET/Ag_2_O NPs samples obtained at different pH.	[46]
18	Ag_2_O-TiO_2_ NPs	50–150	*Escherichia coli*	Bacteriostatic	1.5 mg/mL	The nanocomposite increased photocatalytic degradation of aniline and inhibit *E. coli* growth.	[47]
19	Ag_2_O-TiO_2_ NPs immobilized on doped by cellulose	10 ± 5	-	Proposed bactericidal by photocatalysis	-	The nanocomposite increased photocatalytic degradation of methylene blue, Rhodamine B and norfloxacin under the irradiation of UV light.	[48]
20	Ag_2_O NPs synthesized with culture *Bacillus paramycoides*	28–38	*Enterobacter* sp., *Micrococcus* sp. *Salmonella* sp., *Vibrio parahaemolyticus*	Bactericidal	20 µg/mL	Ag_2_O NPs showed significant bactericidal and antibiofilm activity through bacterial binding. Ag_2_O NPs had cytotoxic action versus A549 cancer cell line.	[49]
21	Precipitated Ag_2_O NPs	30	*Escherichia coli*	Bacteriostatic Bactericidal	30 µg/mL 40 µg/mL	Ag_2_O NPs almost completely inhibited the growth of *E. coli* and caused lysis of bacterial cells.	[50]
22	Green synthesized Ag_2_O NPs with *Lawsonia inermis* extract	~39	*Aspergillus* sp., *Candida albicans,* *Escherichia coli,* *Penicillium* sp., *Pseudomonas aeruginosa,* *Staphylococcus aureus*	Bacteriostatic Fungistatic	23.1 µg/mL * (MIC against *Aspergillus* sp was 0.1 M)	Ag_2_O NPs showed comparable bacteriostatic activity against Gram-positive and Gram-negative bacteria	[51]
23	Borosiloxane Ag_2_O NPs nanocomposite	65	*Escherichia coli*	Bacteriostatic Bactericidal	1 µg/mL	Ag_2_O NPs doped into a borosiloxane matrix pronounced bacteriostatic and bactericidal properties via generation of ROS but did not have cytotoxicity against eukaryotic cells.	[52]
24	PLGA and Ag_2_O NPs nanocomposite	35	*Escherichia coli*	Bacteriostatic Bactericidal	1 µg/mL	Ag_2_O NPs increased generation of H_2_O_2_ and OH-radicals, which can lead to damage to bacterial DNA and proteins but does not have cytotoxicity against mammalian cells.	[53]
25	Ag_2_O NPs in *Bacillus thuringiensis* SSV1 culture supernatant	10–40	*Bacillus cereus,* *Enterococcus faecalis,* *Escherichia coli,* *Proteus mirabilis,* *Pseudomonas* sp., *Staphylococcus aureus*	Bacteriostatic	0.16 µg/mL	“Green synthesized” Ag_2_O NPs shower a weak bacteriostatic effect against both Gram-positive and Gram-negative bacteria. Ag_2_O NPs, but not *B. thuringiensis* induced antimicrobial action.	[54]
26	ZrO_2_-Ag_2_O NPs	14–42	*Bacillus subtilis,* *Streptococcus mutans*, *Escherichia coli,* *Klebsiella oxytoca*, *Pseudomonas aeruginosa,* *Staphylococcus aureus*	Bacteriostatic	0.1 µg/mL	ZrO_2_ NPs enhanced the bacteriostatic effect of Ag_2_O NPs. The bacteriostatic effect of both Ag_2_O NPs and ZrO_2_-Ag_2_O depends more on the bacterial species than on belonging to Gram-positive and Gram-negative bacteria.	[55]
27	Ag_2_O/Ag NPs with *Fusarium oxysporum* components	6–8	*Aspergillus niger,* *Bacillus subtilis*	Bacteriostatic Fungistatic	50 µg/mL	The antibacterial action was realized via increased ROS generation	[56]
28	Ag_2_O NPs conjugated with starch in different proportions	30–110	*Bacillus cereus,* *Escherichia coli,* *Listeria monocytogenes,* *Proteus vulgaris,* *Pseudomonas putida,* *Salmonella typhymurium,* *Staphylococcus aureus,* *Staphylococcus saprophyticus*	Bacteriostatic	100 µg/mL	The bacteriostatic properties of starch-conjugated Ag_2_O NPs enhanced with increasing size and starch/Ag_2_O NPs ratio.	[57]
29	Ag_2_O NPs synthesized by precipitation method	16	*Aeromonas hydrophila* ATCC 7966T	Bacteriostatic	60 µg/mL	Ag_2_O NPs starting at 60 µg/mL inhibited bacterial growth. CFU of *A. hydrophila* was not found on agar at concentrations of Ag_2_O NPs above 240 µg/mL.	[58]
30	Ag and Ag_2_O NPs synthesized by reduction of [Ag(NH3)_2_]^+^ and conjugated by different sugars	25	*Enterococcus faecalis,* *Escherichia coli*, *Staphylococcus aureus,* *Enterococcus faecium*, *Klebsiella pneumonia* ESBL-positive, *Pseudomonas aeruginosa* methicillin-susceptible, *Pseudomonas aeruginosa*, *Staphylococcus aureus* vancomycin-resistant, *Staphylococcus epidermidi* meithicillin-resistant, *Staphylococcus epidermidis* methicillin-resistant	Bacteriostatic Bactericidal	0.68 µg/mL	Ag and Ag_2_O NPs showed more pronounced antimicrobial activity against Gram-negative bacteria. The addition of glucose and lactose to the NP synthesis medium significantly enhanced the antimicrobial effect of NPs.	[59]
31	Ag_2_O and Ag NPs synthesized using *Ficus benghalensis* extract	42.7	*Lactobacilli* sp., *Streptococcus mutans*	Bacteriostatic Bactericidal	100 µg/mL/ 150 µg/mL	Ag_2_O NPs equally inhibited the growth of the studied oral pathogens, regardless of Gram staining. *Ficus benghalensis* extract reduced MIC/MBC by 25% compared to Ag_2_O NPs without extract or silver salt solution	[60]
32	Ag_2_O NPs synthesized using *Nitrobacter* sp. (strain NCIM 5067) extract	40	*Escherichia coli,* *Klebsiella pneumonia,* *Salmonella typhimurium,* *Staphylococcus aureus*	Bacteriostatic	100 µg/mL	Ag_2_O NPs/*Nitrobacter* sp. extract inhibited the growth of both Gram-positive and Gram-negative bacteria equally. The degree of inhibition was comparable to the effects of streptomycin (100 µg/mL). Ag_2_O NPs/*Nitrobacter* sp. extract showed antioxidant properties.	[61]
33	Ag_2_O NPs conjugated with moxifloxacin	49.76	*Aspergillus Niger,* *Bacillus subtilis,* *Candida albicans,* *Escherichia coli,* *Pseudomonas aeruginosa,* *Staphylococcus aureus*	Bacteriostatic Fungistatic	40–60 µg/mL * (initial 40–60 µl of suspension with 0.05 mg/mL)	The conjugation of Ag_2_O NPs with moxifloxacin increased the area of the zone of inhibition for all stufied microorganisms by 2–3 times compared to non-conjugated Ag_2_O NPs. The photocatalytic action is proposed mechanism of antimicrobial action.	[62]
34	Ag_2_O NPs conjugated with silk fibroin (Ag_2_O-SF)	15	*Escherichia coli,* *Mycobacterium tuberculosis,* *Staphylococcus aureus*	Bacteriostatic	115.9 µg/mL * (0.5 mM Ag_2_O)	The conjugation of Ag_2_O NPs with silk fibroin enhances the bacteriostatic properties of Ag_2_O NPs	[63]
35	Ag_2_O NPs composite with *Lippia citriodora* plant powder	20	*Aspergillus aureus,* *Staphylococcus aureus*	Bacteriostatic Fungistatic	0.1 mg/mL	Ag_2_O NPs/*L. citriodora* showed antibacterial and antifungal properties. Antibacterial activity was more pronounced and comparable to the activity of tetracycline. Ag_2_O NPs/*L. citriodora* significantly accelerated wound healing in rats compared to Ag_2_O NPs or controls.	[64]
36	Ag/Ag_2_O NPs with leaf extract of *Eupatorium odoratum*	8.2–20.5	*Bacillus subtilis,* *Candida albicans,* *Escheerichua coli,* *Salmonella typhi,* *Staphylococcus aureus*	Bacteriostatic Fungistatic	25–75 µg/mL 100 µg/mL	Ag_2_O NPs/*E. odoratum* inhibited the growth of Gram-negative bacteria to a greater extent compared with Gram-positive and fungi.	[65]
37	Ag_2_O NPs with *Cleome gynandra* extract	66	*Escheerichua coli,* *Staphylococcus aureus*	Bacteriostatic	~4.2 mg/mL * (20 µl suspension of 0.9 mM AgNO_3_)	Ag_2_O NPs/*C. gynandra* inhibited the growth of Gram-negative bacteria to a greater extent than Gram-positive ones	[66]
38	Highly or poorly oxidized AgO/Ag/SnO_2_	10–20	*Collectotrichum siamense* strains BRSP08 and BRSP09, *Phytophthora cactorum,* *Stenotrophomonas maltophilia,*	Bacteriostatic Fungistatic	0.4 µg/mL * (10 µg/spot, spot is 40 µL)	Nanocomposites with highly oxidized AgO NPs had a more pronounced bacteriostatic effect, and composites of NPs with weakly oxidized AgO NPs had a more pronounced fungistatic effects.	[67]
39	Ag_2_O NPs	17.45	*Bacillus aerius,* *Bacillus circulans,* *Escherichia coli,* *Pseudomonas aeruginosa*	Bacteriostatic Bactericidal	5 µg/mL 7.5 µg/mL	Ag_2_O NPs had a more pronounced antibacterial effect against Gram-negative bacteria compared to Gram-positive ones. The mechanism of antibacterial action is inhibition of ATP synthesis.	[68]
40	Ag_2_O/Ag NPs synthesized in extract *Aloe vera*	10–60	*Candida albicans,* *Candida glabrata,* *Candida parapsilopsis,* *Escherichia coli,* *Staphylococcus aureus*	Bacteriostatic Fungistatic	10 µg/mL	Ag_2_O/Ag NPs/*Aloe vera* inhibited the growth of Gram-negative bacteria to a greater extent than Gram-positive ones. Antimicrobial activity was comparable to 10 µg/mL carbenicillin or ampicillin. Antifungal action depended on the species of fungus. The most effective antimicrobial effect was show against *C. parapsilopsi.*	[69]
41	SrTiO_3_ nanotubes (NTs) embedded with Ag_2_O NTs	10×80	*Staphylococcus aureus*	Bactericidal	―	SrTiO_3_ NTs/Ag_2_O NPs inhibited the growth of *S. aureus*. The antimicrobial effect was realized due to Ag_2_O NPs.	[70]
42	Ag_2_O NPs/Ti NBs	3–10	*Bacillus subtilis*	Bactericidal	100 µg/mL	Ag_2_O/Ti NPs reduced the number of *B. subtilis* CFU compared to the control. Light enhanced the antimicrobial properties of Ag_2_O/Ti NBs.	[71]
43	Ag_2_O NPs/Ti NBs	5–30	*Escherichia coli,* *Staphylococcus aureus*	Bactericidal	1.27 µg/mL	Ag_2_O NPs/Ti NBs killed 100% during 14–21 days. The release of Ag^+^ is the mechanism of its antibacterial action.	[72]
44	Ag_2_O/Ag NPs synthesized in *Vaccinium arctostaphylos* extract	7–10	*Bacillus subtilis,* *Escherichia coli,* *Salmonella enteritidis,* *Staphylococcus aureus*	Bacteriostatic	<116 µg/mL * (amount of NPs synthesized from 1 mM of AgNO_3_)	The antimicrobial effect against Gram-positive bacteria is more pronounced than against Gram-negative ones.	[73]
45	Ag_2_O NPs with polyhedral shape	400–700	*Escherichia coli*	Bactericidal	10 µg/mL	The antimicrobial effect of cubic NPs is two times higher than that of octahedral NPs.	[74]
46	H_2_Ti_3_O_7_•2H_2_O/Ag_2_O NPs nanocomposites	10–40	*Escherichia coli,* *Bacillus subtilis*	Bacteriostatic Bactericidal	25 µg/mL 50 µg/mL	The addition of Ag_2_O NPs to H_2_Ti_3_O_7_·2H_2_O increased the antimicrobial properties. The antibacterial action was equal against Gram-negative and Gram-positive bacteria.	[75]
47	Ag/AgO/Ag_2_O NPs/*Coleus aromaticus* extract/reduced graphene oxide	2–4	*Escherichia coli,* *Klebsiella pneumonia,* *Staphylococcus aureus*	Bacteriostatic	50 mg/mL	Ag/AgO/Ag_2_O NPs improved antimicrobial properties of resulting composite. The bacteriostatic effect against Gram-positive or Gram-negative bacteria was comparable.	[76]
48	Ceftriaxone/Ag_2_O NPs	35.54	*Escherichia coli*	Bacteriostatic Bactericidal	10 µg/mL	The antimicrobial activities of ceftriaxone and Ag_2_O NPs, assessed by zones of inhibition, were summarized.	[77]
49	Ag/Ag_2_O NPs synthesized in *Pseudomonas aeruginosa* M6 extract without cells	~10.4	*Escherichia coli,* *Pseudomonas aeruginosa,* *Staphylococcus aureus,* *Candida albicans,* *Candida glabrata,* *Mycobacterium smegmatis*	Bacteriostatic Fungistatic	<12 µg/mL * (100 µL suspension of *P. aeruginosa* M6 in 1 mM AgNO_3_/ml)	Antibacterial and antifungal activity significantly depended on the species of microorganisms. Interspecies differences in antibacterial action are more pronounced than differences between Gram-positive and Gram-negative bacteria.	[78]
50	Ag/Ag_2_O NPs synthesized in cell-free extract of *Kitasatospora albolonga* fungi	20	*Pseudomonas aeruginosa* multi drug resistant	Bacteriostatic	125 µg/mL	Ag/Ag_2_O NPs had bacteriostatic effect and enhanced the antibacterial effect of 800 µg/mL carbenicillin.	[79]
51	Ag/Ag_2_O NPs synthesized in dead yeast *Rhodotorula mucilaginosa* biomass	11	*Cryptococcus neoformans,* *Escherichia coli* multi-drug resistant, *Staphylococcus aureus*	Bacteriostatic Bactericidal Fungistatic Fungicidal	2 µg/mL 5 µg/mL 0.2 µg/mL 0.2 µg/mL	Ag/Ag_2_O NPs/*R. mucilaginosa* showed significant antibacterial and antifungal activity and moderate cytotoxicity against eukaryotic cell lines. Cytotoxic concentrations were 4–10 times higher than antimicrobial ones. NPs can be considered as a possible agent for the treatment of oncology.	[80]
52	Ag/Ag_2_O NPs synthesized in silver films under oxygen plasma treatment	6–38	*Staphylococcus aureus*	Bacteriostatic	―	The most bacteriostatic effect was shown by Ag_2_O NPs with smallest size. This NP were obtained at plasma power of 1250 W.	[81]
53	Ag_2_O NPs and nano-rod complex (1), [Ag (3-bpdh)(NO_3_)]_n_	45–60	*Enterococcus faecalis,* *Escherichia coli,* *Pseudomonas aeruginosa,* *Staphylococcus aureus*	Bacteriostatic	6.25–25 µg/mL	Ag_2_O NPs were equally effective against Gram-positive and Gram-negative bacteria. Least bacteriostatic effect against *Escherichia coli* (PTCC1330) was shown.	[82]
54	Ag_2_O NPs mixed with *Centella Asiatica* or *Tridax* sp. leaf powder	11–12	*Aspergillus aureus,* *Aspergillus fumigates,* *Staphylococcus aureus,* *Staphylococcus epidermidis*	Bacteriostatic Fungistatic	100 µg/mL	Ag_2_O NPs/*Tridax* had a more pronounced antimicrobial effect than Ag_2_O NPs/*Centella*. The mechanism of toxicity is photocatalytic activity.	[83]
55	Ag/Ag_2_O NPs synthesized in *Hylocereus undatus* extract	25–26	*Escherichia coli,* *Pseudomonas aeruginosa,* *Staphylococus aureus*	Bacteriostatic	500 µg/mL	Ag_2_O NPs/*H. undatus* showed more strong bacteriostatic action against Gram-positive bacteria than against Gram-negative bacteria.	[84]
56	Ag_2_O NPs synthesized in *Telfairia occidentalis* extract	8–10	*Klebsiella pneumoniae*	Bacteriostatic	10 µg/mL	Ag_2_O NPs/*T. occidentalis* had persistent dose-dependent bacteriostatic effect.	[85]
57	Ag_2_O NPs with addition of 1–9% Sr	35.7–48.4	*Enterobacter aerogens,* *Bordetella bronchiseptaca,* *Salmonella typhimurim,* *Aspergillus fumigatus,* *Aspergillus niger,* *Fusarium soloni*	Bacteriostatic Fungistatic	~100 µg/mL (100 μg/disc)	3% Sr/Ag_2_O NPs showed maximal bacteriostatic and fungistatic activities. Antibacterial activity did not depend on species. Antifungal activity was species dependent.	[86]
58	Ag_2_O/Ag NPs synthesized by precipitation of AgNO_3_ in N-propanol	19–60	*Bacillus cereus,* *Candida albicans* *Chlorella vulgaris,* *Enterococcus faecalis,* *Pseudomonas aeruginosa,* *Salmonella typhimurium,* *Staphylococcus aureus*	Bacteriostatic Fungistatic	5 µg/mL	Ag_2_O/Ag NPs inhibited growth of all studied microbes, had anti-biofilm activity. Mechanism of toxicity is Ag^+^ releasing. Ag_2_O/Ag NPs showed less cytotoxicity against Vero cell line than equal amount of AgNO_3_.	[87]

*—concentration is not directly indicated in article in µg/mL and is calculated based on description in Materials and Method sections. Original data are shown in brackets.

## Data Availability

Data sharing not applicable.
